# Aaron (Tim) Beck, MD

**DOI:** 10.1192/bjb.2022.2

**Published:** 2022-10

**Authors:** Judith Beck, Sarah Fleming

Formerly Professor Emeritus in Psychiatry, University of Pennsylvania, President Emeritus, Beck Institute for Cognitive Behavior Therapy, Pennsylvania, USA

**Figure:**
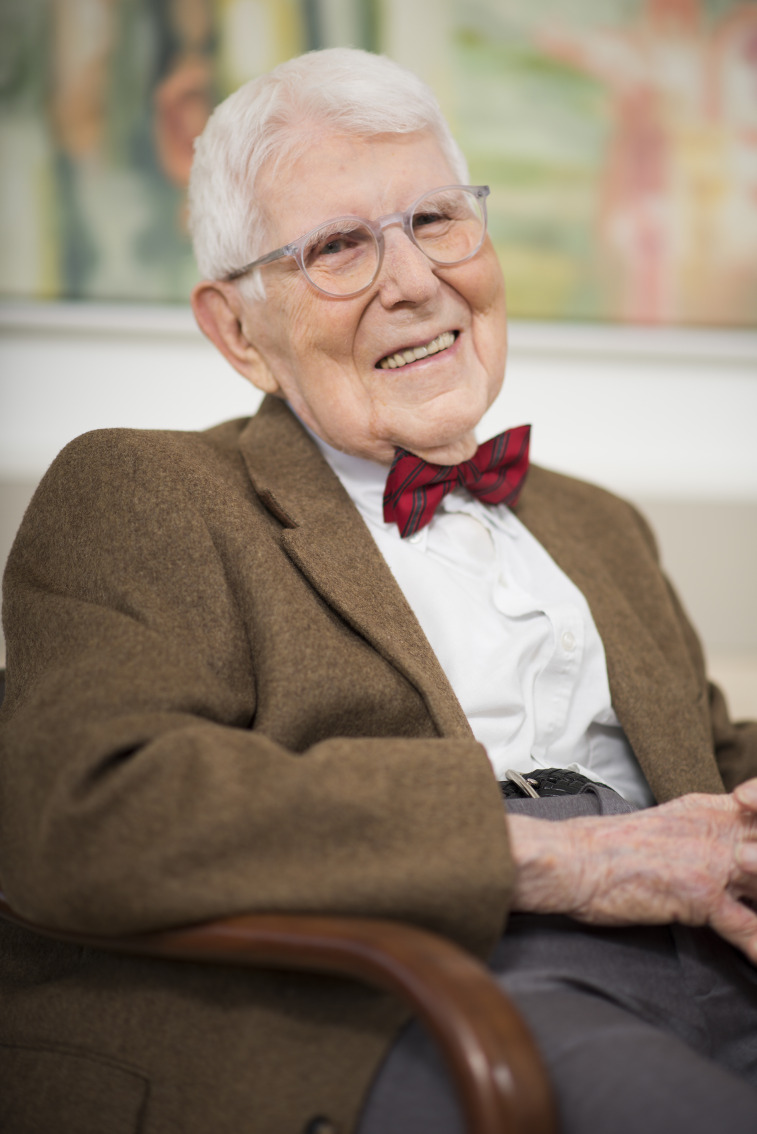

Aaron (Tim) Beck, universally regarded as the originator of cognitive–behavioural therapy (CBT), died aged 100 on 1 November 2021. From the 1940s to the 1960s, psychoanalysis was undisputed in the USA as the dominant explanatory theory for all mental disorders. Further, psychoanalytic psychotherapy was the treatment of choice for most, if not all mental illnesses. Psychoanalysis held a significant but less commanding position in Britain and in Western Europe, where neuropsychiatry was a competing force.

In the early 1960s, Beck began to publish articles both sceptical of the validity of psychoanalytic theory and proposing a new way of explaining and treating psychiatric disorders, particularly depression. He had undergone a full training as a psychoanalyst and recognised the importance of experimentally validating psychoanalytic constructs so psychoanalysis would be accepted in the scientific world. The experiments he conducted, however, failed to validate psychoanalytic theories of depression. He began to question whether negative thoughts described by patients were a consequence of intrapsychic conflicts and whether these thoughts would disappear if one could resolve these conflicts. Further, Beck found no evidence for the psychoanalytic hypothesis that depression arose from the patient's own hostility turned inwards.

Stimulated by what he learned from his patients, Beck took the view that negative thoughts were part and parcel of mental disorders. He described these negative thoughts as forming what he called ‘a cognitive triad’ in which patients experienced maladaptive cognitions about themselves, the world, and their future. He characterised these thoughts as ‘automatic’. As a result of distorted thinking reflecting deeply held assumptions, they seemed to enter patients’ minds spontaneously as interpretations of their internal and external experiences. If one could modify these inaccurate or unhelpful thoughts by encouraging patients to question them, then patients could more easily change their dysfunctional behaviours, solve current problems and feel better emotionally. Their depressive illnesses would be relieved.

Most unusually for a psychotherapist at that time, Beck believed that psychotherapy could be scientifically evaluated. His development of the Beck Depression Inventory and other measurement tools opened the way for quantifiable assessment. In 1977, his team published the first controlled trial of cognitive therapy (as CBT was then called) comparing its efficacy with that of antidepressant medication.^1^ During the 1980s and 1990s, CBT largely replaced psychoanalysis as the dominant psychological theory for the explanation and treatment not just of depression but of most mental disorders. CBT principles were adapted for use in the treatment of anxiety, obsessional and eating disorders as well as a wide range of other conditions. More recently it has been further adapted for use in addiction disorder, pain syndromes and with schizophrenic delusions. It has been applied in a variety of settings, including community mental health, in-patient hospitals, out-patient clinics, schools and forensic settings, for adults, older adults, children, couples and families from a range of backgrounds.

Working alongside David Clark, Beck spent several periods in England in the Department of Psychiatry at the University of Oxford, and CBT became particularly influential in Britain. Its principles underlie the psychological therapies delivered in the Improving Access to Psychological Therapies (IAPT) programme. Although not without its critics, IAPT remains widely applied. Beck himself continued to take part in new developments, especially recovery-oriented cognitive therapy (CT-R) for individuals diagnosed with serious mental health conditions, until his last days.

Aaron Temkin Beck, known as Tim, was born in Providence, Rhode Island, on 18 July 1921, the youngest of four children. His parents, Elizabeth Temkin and Harry Beck, a printer, were Jewish immigrants from Ukraine. He graduated from Brown University in 1942 with a BA in English and Political Science, and in 1946 he received his medical degree from the Yale School of Medicine. In 1954, Beck began teaching psychiatry at the University of Pennsylvania School of Medicine, where he would remain for the duration of his career. He received his psychoanalytic training at the Philadelphia Institute but, having completed training, because of his unorthodox views and clinical practice, was rejected for certification by the American Psychoanalytic Institute.

Throughout his 70-year career, Dr Beck authored or co-authored 25 books and over 600 scientific articles. He received more than 50 awards for his research and contributions to the field of medicine, including the Heinz Award for the Human Condition in 2001 and the Albert Lasker Award for Clinical Medical Research in 2006. He received honorary degrees from Yale University, the University of Pennsylvania and Brown University. He is also known for his career-long advancement of evidence-based treatment and the development of instruments used in patient assessment and monitoring. He made a further major contribution to the classification and prevention of suicide. He was instrumental in 1994 in co-founding, with his daughter Judith, the Beck Institute for Cognitive Behavior Therapy.

Beck was an enthusiastic teacher and mentor to countless junior colleagues. Many of his trainees and professional colleagues enjoyed rich friendships with him and he kept in touch with hundreds of clinicians, educators and researchers, offering advice and support both professionally and personally. He was known for his positive attitude, even as he suffered from physical challenges as he aged. He had a wide range of interests, ranging from the natural and social sciences to law, politics, sports and pop culture.

Beck is survived by his wife Phyllis, whom he married in 1950, four children, ten grandchildren and ten great-grandchildren.
